# An Unusual Case of Morphea in the Setting of Aplastic Anemia

**DOI:** 10.7759/cureus.7562

**Published:** 2020-04-06

**Authors:** Sierra Mastrantonio, Brian R Hinds, Jeremy A Schneider, Rachel Sennett, David G Cotter

**Affiliations:** 1 Department of Dermatology, University of Nevada Las Vegas School of Medicine, Las Vegas, USA; 2 Department of Dermatology, University of California San Diego, San Diego, USA

**Keywords:** morphea, aplastic anemia, scleroderma, systemic sclerosis, multiple autoimmune syndrome, lichen sclerosus

## Abstract

Cutaneous sclerosis occurs in association with a variety of systemic diseases, including hematologic malignancy, plasma cell dyscrasias, solid organ tumors, and other systemic autoimmune conditions. Herein, we present a unique case of morphea/lichen sclerosus overlap arising in association with aplastic anemia. To expand upon this rare case, we also review the literature surrounding paraneoplastic sclerosing skin disorders. A 53-year-old man presented with a 13-month history of progressive and generalized skin changes. Exam revealed irregular, hypopigmented indurated plaques with focal areas of scale on the bilateral axillae and hips, as well as hyperpigmented brown papules and plaques on the back. Laboratory evaluation revealed pancytopenia and positive anti-nuclear antibody (1:160). Bone marrow biopsy demonstrated hypocellular marrow consistent with aplastic anemia. Furthermore, skin biopsies revealed lichen sclerosus overlying superficial morphea, consistent with a paraneoplastic sclerodermoid-like eruption. While preparations for hematologic-directed therapies were made, skin-directed therapy with a combination topical steroids and topical calcineurin inhibitors was initiated. Eosinophilic fasciitis and scleroderma have been linked to aplastic anemia, and herein, we expand upon this phenomenon by presenting our case of generalized plaque morphea/lichen sclerosus overlap arising in the setting of aplastic anemia. Dermatologists must be aware of this rare association in order to identify precocious hematologic disease.

## Introduction

Sclerosing skin disorders comprise a broad category of disease and encompass multiple distinct autoimmune fibrosing disorders including diffuse and limited systemic sclerosis (scleroderma), morphea (localized scleroderma), and lichen sclerosus, among others [1]. Each diagnosis is defined by the extent of cutaneous and/or visceral involvement, as well as the presence of specific auto-antibodies and the histologic changes in skin architecture. While the precise mechanisms underlying the pathogenesis of these diseases remain largely unknown, their impact is well established. Systemic sclerosis causes extensive microvascular damage throughout the skin and internal organs via excessive collagen deposition, with correspondingly significant effects on morbidity and mortality [1]. By contrast, morphea has fibrosis limited to the skin, subcutaneous tissue, and bone, but nonetheless may be life-altering, as it can result in joint contractures, growth restriction, and prominent craniofacial disfigurement [1]. A third subset of disorders has also been identified and designated “scleroderma-like” [1]. This category shares the characteristic of cutaneous fibrosis, but the underlying cause and subsequent potential for organ involvement is much more variable [1]. Scleroderma-like eruptions have been associated with a variety of systemic diseases, including hematologic malignancy, plasma cell dyscrasias, solid organ tumors, and other systemic autoimmune conditions [1].

The widespread nature of systemic sclerosis contributes to its higher mortality rate [1]. As part of the European League Against Rheumatism (EULAR) Scleroderma Trial and Research (EUSTAR), the highest predictors for mortality in systemic sclerosis were identified as age at diagnosis, the degree of fibrotic renal involvement, pulmonary involvement/complications, and the presence of skin induration [2]. Systemic sclerosis has also been associated with an increased risk for solid organ tumors in many of the same organ systems that predict disease mortality. Most commonly, systemic sclerosis has been correlated with cancers of the bladder, lung, liver, and skin [3-5].

Hematologic malignancy and plasma cell dyscrasias are additional conditions observed in association with systemic sclerosis and/or other scleroderma-like eruptions [6]. Specifically, patients with systemic sclerosis exhibit a significantly higher incidence of leukemia [3,7]. Research suggests that systemic sclerosis may, in fact, arise as a paraneoplastic phenomenon in patients with malignancy via autoimmune mechanisms [4-5]. Moreover, patients with plasma cell dyscrasias such as multiple myeloma can manifest similar paraneoplastic scleroderma-like tissue reactions, including eosinophilic fasciitis, morphea, and systemic sclerosis [8]. Other hematologic aberrancies arising independent of myeloid and lymphoid lineages, such as autoimmune thrombocytopenic purpura, have also been linked to scleroderma and morphea through possible autoimmune mechanisms [9-10]. The concomitant presentation of hematologic dyscrasias with sclerosing skin disorders raises interesting questions as to a possible pathogenic link underlying these seemingly disparate conditions, but in general, the association of these diagnoses is not yet well understood.

One hematologic disease rarely associated with scleroderma-like eruptions is aplastic anemia. The first reported case of pancytopenia in association with systemic sclerosis was documented in the 1976 British Medical Journal [11]. To the best of our knowledge, no case of aplastic anemia arising in association with morphea or lichen sclerosus has been reported [6,12]. Herein, we present a unique case of morphea/lichen sclerosus overlap arising in association with aplastic anemia and review the literature surrounding paraneoplastic sclerosing skin disorders.

## Case presentation

A 53-year-old man was transferred to a Bone and Marrow Transplantation (BMT) service for work-up and treatment of pancytopenia. Upon transfer, the dermatology consult service was called to evaluate a “hypopigmented rash”. The patient first noticed skin changes on his hips approximately 13 months prior to admission. Within two months, his eruption had spread to involve the bilateral axillae. He recalled a brief time in which the skin on his hips and axillae was red, but he denied scaling, pruritus, or pain. In this same earlier time period, results of a complete blood count and a complete metabolic panel had been within normal limits. A trial of topical triamcinolone 0.01% cream at that time provided no relief for his rash.

Upon transfer to the BMT service, his symptoms included fatigue, ongoing skin changes, a feeling of “tightening” around joints that limited his range of motion, and both inguinal and submandibular lymphadenopathy. A complete review of systems was notable for intermittent episodes of hematochezia and melena. His past medical history was notable for rheumatoid arthritis requiring multiple orthopedic procedures. He denied any personal or family history of prior skin diseases and had no personal history of malignancy. His only medications were omeprazole and vitamin D. His social and family histories were noncontributory. 

**Figure 1:**
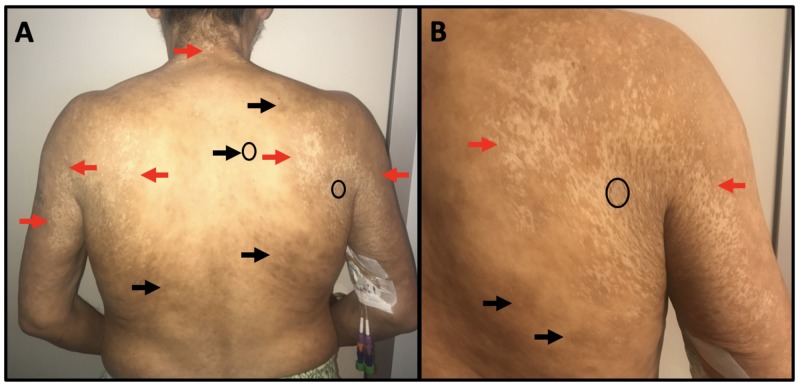
Physical exam findings All red arrows indicate shiny, hypopigmented and indurated sclerotic papules coalescing into plaques with focal scale. Black arrows indicate hyperpigmented, indurated papules and plaques. A: Skin exam findings on the patient’s back and posterior axillae. Black ovals delineate two areas where punch biopsies were obtained on the right paraspinal skin and the right posterior axillary skin. B: Skin exam findings on the patient’s right posterior axillae. The black oval delineates the right posterior axillary biopsy site.

**Figure 2:**
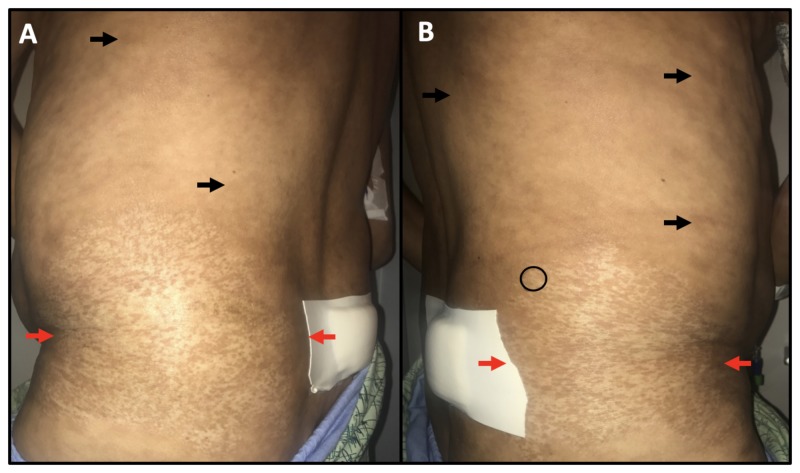
Physical exam findings continued Between the red arrows are shiny, indurated sclerotic papules that coalesce into reticular and dyschromic plaques with focal scale. Black arrows indicate hyperpigmented, indurated papules and plaques. The bandage overlies the patient’s bone marrow biopsy site. A: Skin exam findings on the patient’s left flank. B: Skin exam findings on the patient’s right flank. Black oval delineates the right flank skin biopsy site.

Physical exam was remarkable for extensive inguinal, axillary, and cervical adenopathy as well as marked skin changes on the trunk, extremities, axillae, and hips. Skin examination revealed shiny, hypopigmented and indurated papules coalescing into irregular, hypopigmented sclerotic plaques with focal areas of scale on the posterior neck, peri-axillary skin, and hips as well as hyperpigmented, indurated papules and plaques on the back (Figure 1 and Figure 2). To establish a diagnosis, the shiny, sclerotic axillary, and flank plaques, as well as the indurated, truncal hyperpigmented papules and plaques, three punch biopsies were obtained. Specimens were collected from the right flank, right posterior axillary skin, and right paraspinal skin and encompassed two hypopigmented sites (Figure 1B and Figure 2B, black ovals) and one hyperpigmented site (Figure 1A, black oval).

**Figure 3:**
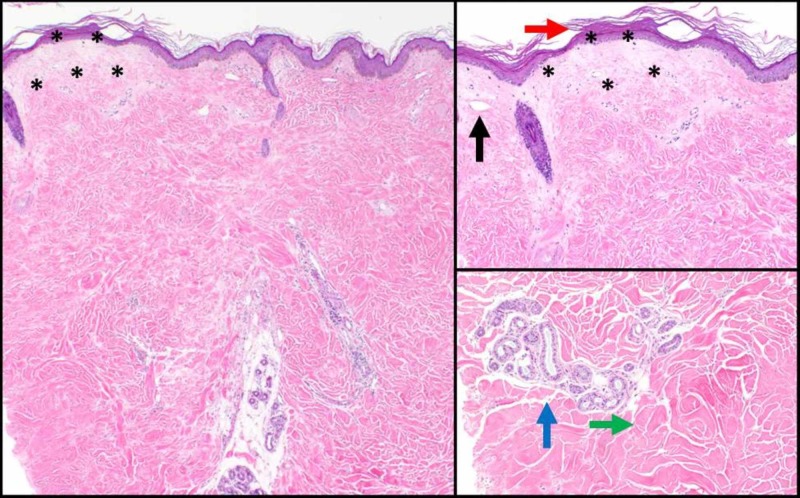
Histologic findings consistent with lichen sclerosus overlying superficial morphea A: Representative cross section of the skin punch biopsy stained with hematoxylin and eosin (H&E) at 4x magnification. B: Skin punch biopsy with H&E stain; 10x. Red arrow indicates hyperkeratosis. Black asterisks surround areas of papillary dermal pallor, and a black arrow indicates an ectatic superficial vessel. C: Skin punch biopsy with H&E stain; 20x. Green arrow indicates thickened collagen bundles. Blue arrow indicates apocrine glands that have lost their normal periadnexal adipose tissue.

All three biopsies shared overlapping histologic features consistent with lichen sclerosus overlying superficial morphea (Figure 3). The biopsy specimens revealed hyperkeratosis (Figure 3B), pallor of the papillary dermis, and ectatic superficial vessels (Figure 3B) consistent with lichen sclerosus. The tissue specimens also showed thickened collagen bundles (Figure 3C) and loss of periadnexal adipose tissue (black arrow; Figure 3C) throughout the specimen, consistent with morphea (Figure 3).

Bloodwork obtained during admission confirmed pancytopenia and revealed elevated lactate dehydrogenase and a positive anti-nuclear antibody (1:160), but negative anti-Scl-70 (Table 1). Bone marrow biopsy showed hypocellular marrow consistent with aplastic anemia. Flow cytometry did not show any evidence of increased or aberrant immune cells. Computed tomography (CT) scans of the chest, abdomen, and pelvis showed axillary and inguinal lymphadenopathy, an adrenal adenoma, and a hepatic cyst.

**Table 1:** Significant laboratory findings IgG, Immunoglobulin G; Scl-70, topoisomerase I; dsDNA, double-stranded DNA; SSA, Sjogren's Syndrome Related Antigen A; SSB, Sjogren's Syndrome Related Antigen B; L, liter; dL; deciliter; ul, microliter

Laboratory Test	Patient’s Results	Normal Range (per American College of Physicians/ACP Guidelines)
White Blood Cell Count (WBC)	0.8 x 10^9^/L	4.0-10 x 10^9^/L
Absolute Neutrophil Count (ANC)	0.1 x 10^9^/L	1.78-5.38 x 10^9^/L (male)
Hemoglobin (Hgb)	7.4 g/dL	14-17 g/dL (male)
Hematocrit (Hct)	20.2%	41-51% (male)
Platelets	26,000 /mL	150,000-350,000 /mL
Lactate Dehydrogenase (LDH)	237 units/L	60-100 units/L
Complete Metabolic Panel (CMP)	All values were within normal limits	---
Coagulation Panel	All values were within normal limits	---
Cytomegalovirus immunoglobulin	Negative	Negative
Mononucleosis	Negative	Negative
Parvovirus B19	Negative	Negative
Hepatitis B virology	Non-reactive	Non-reactive
Hepatitis C virology	Negative	Negative
Human immunodeficiency virus (HIV)	Negative	Negative
Syphilis RPR	Non-reactive	Non-reactive
Anti-nuclear Antibody (ANA)	Positive 1:160	Negative
Scl-70 Antibody	Negative	Negative
dsDNA Antibody	Negative	Negative
Smith Antibody	Negative	Negative
SSA/SSB Antibody	Negative	Negative
Blood Cultures	No growth	No growth

The patient was started on clobetasol 0.05% ointment topically twice daily for two weeks, followed by tacrolimus 0.1% ointment topically twice daily. Following discharge, the patient elected to follow up at a hospital closer to his home for management of his idiopathic aplastic anemia and morphea/ lichen sclerosus overlap.

## Discussion

Aplastic anemia can adopt many clinical presentations, including a rare association with cutaneous sclerosis, specifically, systemic scleroderma or eosinophilic fasciitis [6,12]. Interestingly, an association of aplastic anemia with non-eosinophilic localized scleroderma (morphea) has not yet been reported.

Morphea and its subtypes encompass a complex collection of autoimmune diseases with myriad presentations [13]. Patients who present with generalized and mixed morphea subtypes are considered candidates for systemic immunosuppressive therapy and comprehensive workup due to the high incidence of other concomitant autoimmune syndromes [13]. Autoimmune diseases that are comorbid with morphea vary greatly but include conditions such as autoimmune thrombocytopenic purpura, central nervous system vasculitis, and autoimmune hypothyroidism [9-10].

Our patient presented with skin changes, generalized fatigue, and “joint tightness.” Physical examination was most notable for irregular, dyschromic, and sclerotic plaques on the patient’s bilateral flanks, peri-axillary skin, antecubital fossae, nape of neck, and right groin. Initial workup found the patient to be pancytopenic, with a bone marrow biopsy confirming aplastic anemia. Cutaneous findings were significant for superficial morphea and lichen sclerosus without evidence of eosinophilia, consistent with a paraneoplastic scleroderma-like eruption. This case report highlights an interesting and important association between aplastic anemia and sclerosing skin disorders.

When evaluating the mechanisms of morphea and aplastic anemia, it is important to consider an underlying systemic sclerotic or fibrotic process that may affect both cutaneous and hematologic compartments. The molecular targets of such fibrosis are under investigation [14-15]. Specifically, alterations in molecular pathways such as Wnts, transforming growth factor-b (TGF-b) signaling, and Toll-like receptors (TLRs) are thought to play an important role in driving aberrant fibroblast function underlying localized and systemic sclerosing disorders and represent putative targets for therapeutic intervention [14-15].

At this time, treatment for widespread fibrosis mainly consists of generalized immunosuppressive therapy or bone marrow transplantation, with the latter option only recently approved for cutaneous conditions. In 2018, the American Society for Blood and Marrow Transplantation released a position statement advocating for hematopoietic stem cell transplantation for the management of systemic sclerosis [16]. The position statement cites evidence finding marked improvement in both cutaneous symptoms and solid organ fibrosis in trial patients after receiving a bone marrow transplant, compared to non-transplanted controls [16-17]. Concomitantly, immunosuppressive therapies traditionally used to treat aplastic anemia, such as systemic cyclosporine and anti-thymocyte globulin, have been shown to successfully treat paraneoplastic scleroderma, pansclerotic morphea, and cases of primary systemic sclerosis [18-20]. Together, these findings further suggest commonalities among the pathogenesis of sclerosing skin and bone marrow disorders.

## Conclusions

This case features the presentation of morphea/lichen sclerosus overlap as a paraneoplastic scleroderma-like eruption in the setting of aplastic anemia. Our patient's cutaneous manifestations preceded his aplastic anemia, which may be seen in some patients. We believe a potential underlying pan-fibrotic or pan-sclerotic process affecting both the bone marrow and skin occurred in this patient. For many patients, including the patient presented herein, identification of therapies that can simultaneous address cutaneous and visceral manifestation of sclerosing diseases is paramount. While additional consideration is needed to fully elucidate the pathogenic mechanisms at play and to define the optimal treatments, this case demonstrates the importance of this disease association.
